# Takotsubo cardiomyopathy complicating acute pancreatitis: a case report

**DOI:** 10.1186/s12876-021-01725-5

**Published:** 2021-03-23

**Authors:** John Yeh, James Carroll

**Affiliations:** Institute of Surgery, Townsville Hospital and Health Service, 100 Angus Smith Drive, Douglas, Townsville, QLD 4814 Australia

**Keywords:** Acute alcoholic pancreatitis, Takotsubo cardiomyopathy, Case report

## Abstract

**Background:**

Acute pancreatitis as a trigger of Takotsubo cardiomyopathy has been infrequently described in the literature. Misdiagnosis of this phenomenon can often occur due to overlap in symptomology, particularly in those outside of the usual patient demographic.

**Case presentation:**

A 27-year-old man with a history of alcohol abuse presented with epigastric and chest pain. Electrocardiography showed ischemic changes, and laboratory workup revealed elevated lipase and troponin. He was diagnosed with acute pancreatitis and managed presumptively as acute coronary syndrome. Subsequent coronary angiography was negative for obstructive coronary artery disease, and left ventriculography demonstrated basal hyperkinesis and apical akinesis, characteristic of Takotsubo cardiomyopathy.

**Conclusions:**

Takotsubo cardiomyopathy is a rare complication of acute pancreatitis. Increased awareness of this phenomenon is required to prevent delays in diagnosis and avoid unnecessary interventions and complications.

## Background

Takotsubo cardiomyopathy (TCM) has increasingly gained international awareness since it was first introduced in 1990 [1]. Whilst there are many well-documented triggers of TCM, the role of acute pancreatitis has been only sporadically mentioned in the literature. Increasing awareness of this phenomenon particularly in those outside of the usual patient demographic may lead to earlier diagnosis and avoid unnecessary interventions. We present a rare case of TCM complicating acute alcoholic pancreatitis.

## Case presentation

A 27-year-old Indigenous man, with a history of smoking and no other cardiac risk factors, developed epigastric pain associated with nausea, vomiting and diaphoresis, following significant alcohol consumption the night prior. In the subsequent hours he also developed chest pain. On presentation he was tachycardic to 110 beats per minute, febrile to 38.4 °C, and had epigastric tenderness.

**Fig. 1:**
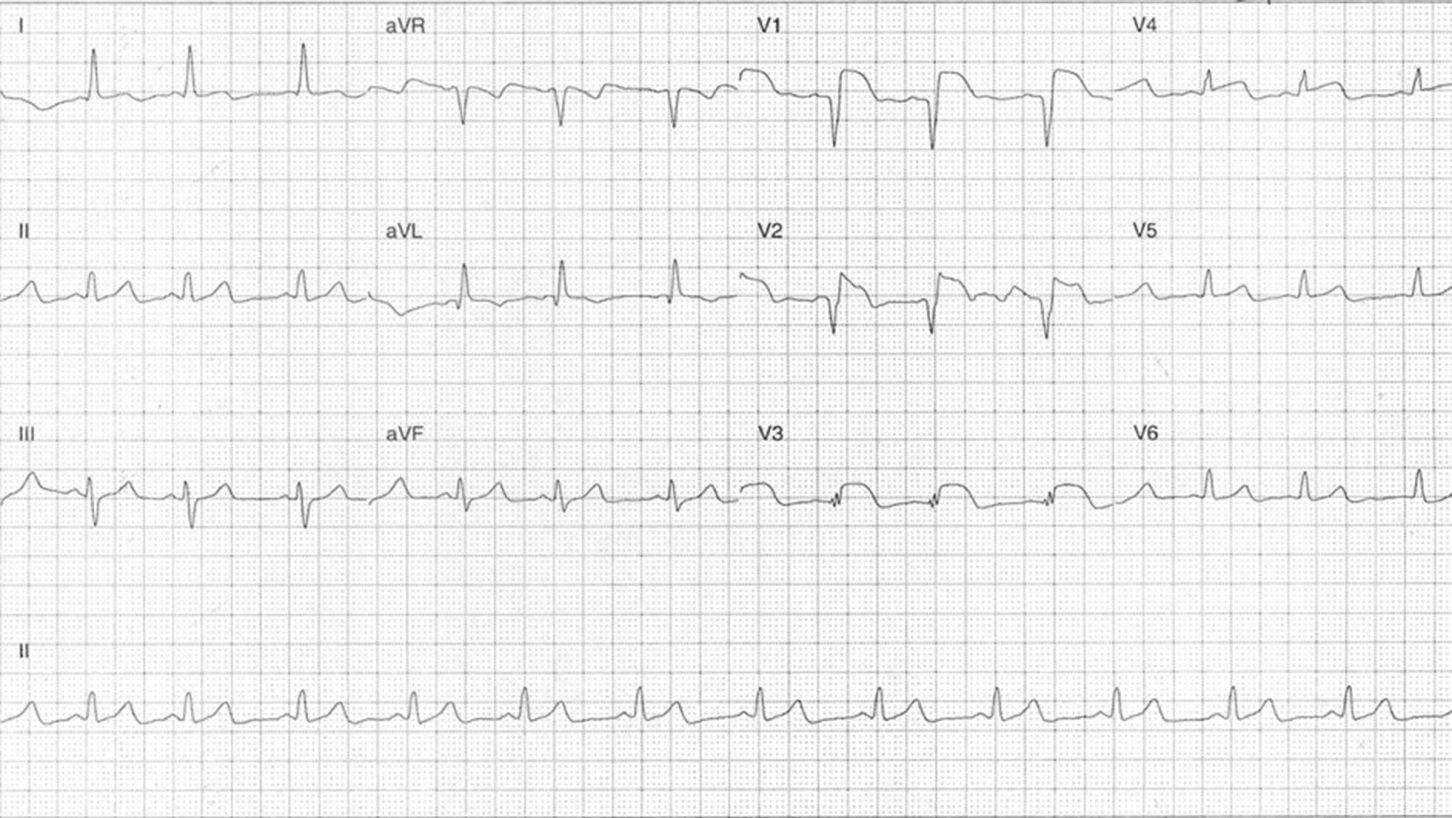
Electrocardiography. ST elevation in leads V1–V3

His laboratory workup showed an elevated lipase of 1310 U/L, leukocytosis of 17.2 × 10^9^/L, C-reactive protein of 26 mg/L, and marginally deranged liver function tests with bilirubin of 26 µmol/L, alkaline phosphatase of 118 U/L, gamma-glutamyl transpeptidase of 101 U/L, alanine aminotransferase of 109 U/L, and aspartate aminotransferase of 141 U/L. Kidney function was maintained with a creatinine of 76 µmol/L, and his lipid profile showed only raised triglycerides at 2.5 mmol/L. His serial troponins rose from 0.77 ng/mL to 1019.63 ng/mL, and electrocardiography (ECG) revealed ST elevation in the anterior leads (Fig. 1). A provisional diagnosis of anterior ST-elevation myocardial infarction was made. The patient was thrombolysed, commenced on dual antiplatelet therapy and low-molecular weight heparin, and transferred to the Coronary Care Unit of the closest tertiary centre.

**Fig. 2:**
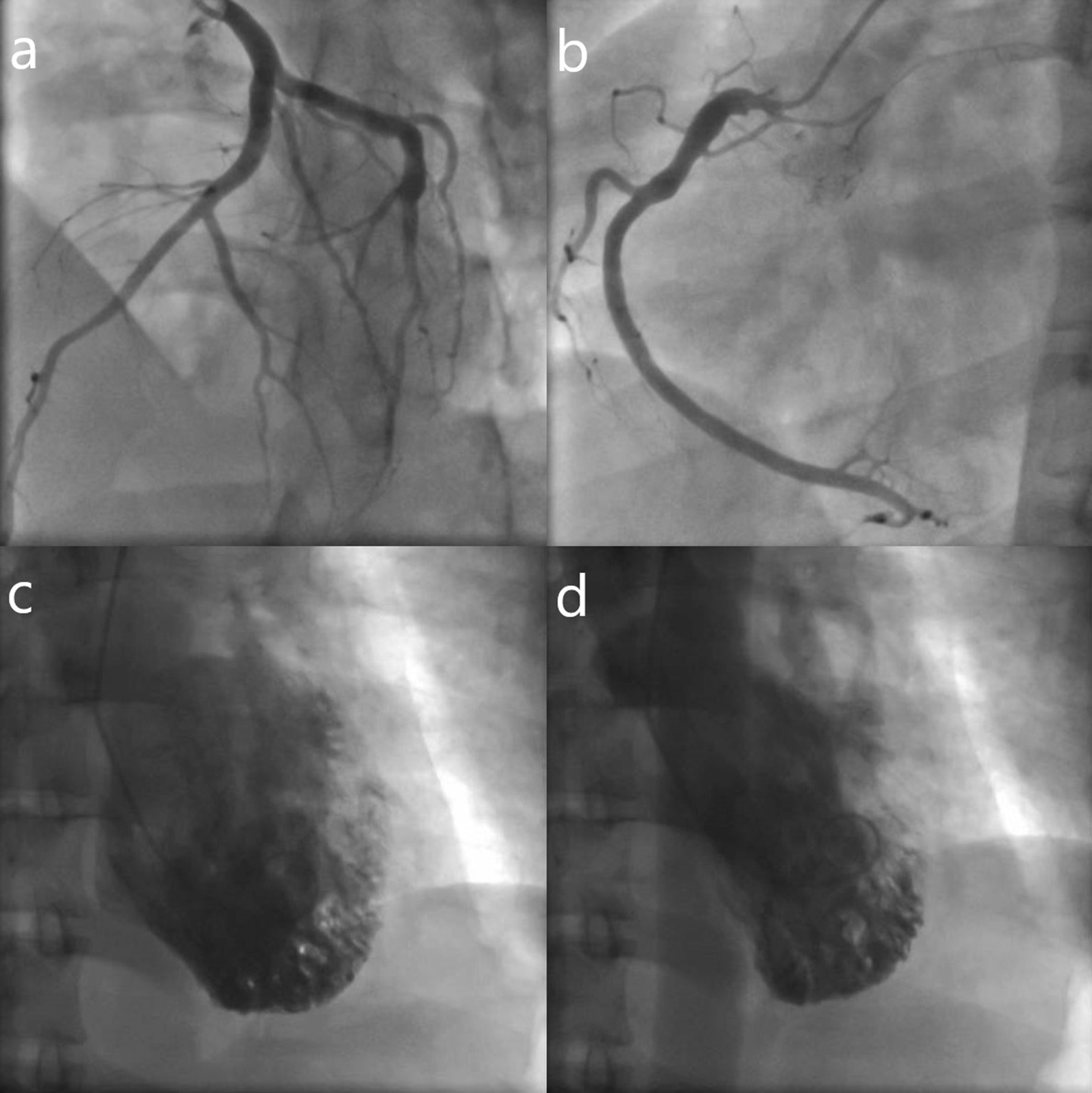
Coronary angiography with left ventriculography. Non-obstructive disease of left (**a**) and right (**b**) coronary arteries. Left ventricle in (**c**) diastole and (**d**) systole demonstrating apical akinesis and basal hyperkinesis

Repeat ECG showed partial resolution of ST-elevation and the presence of Q waves. Transthoracic echocardiography demonstrated severely reduced left ventricular systolic function, with an estimated ejection fraction of 20%. Subsequent coronary angiography with ventriculography showed non-obstructive coronary artery disease, and basal hyperkinesis with apical akinesis of the left ventricle (Fig. 2). The final diagnosis of TCM was made. Dual antiplatelet therapy was ceased, and the patient was commenced on an angiotensin-converting enzyme inhibitor (ACEi) and beta-blocker.

**Fig. 3:**
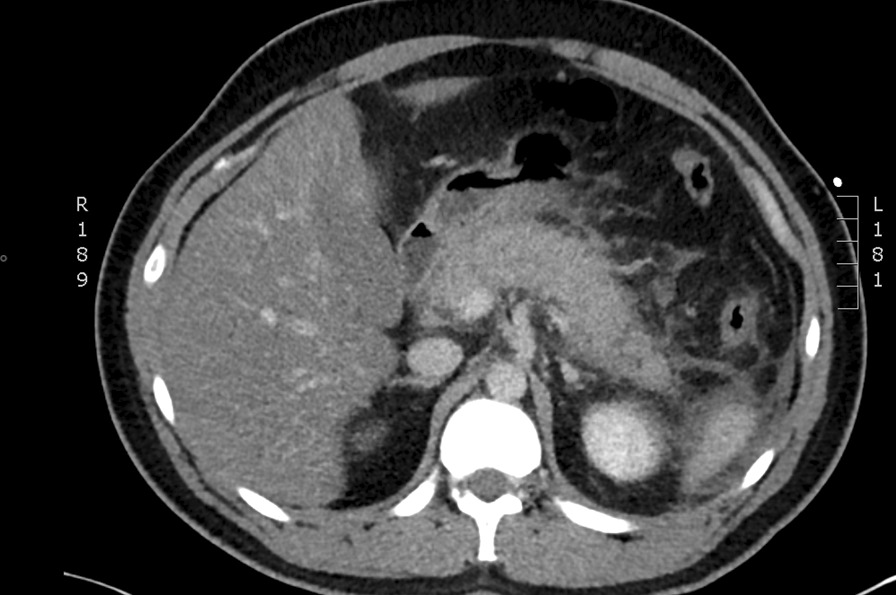
Abdominal computed tomography. Pancreatic oedema with surrounding fat stranding

Meanwhile, hepatobiliary ultrasound was unrevealing for cholelithiasis or choledocholithiasis, and computed tomography of the abdomen demonstrated an oedematous pancreas with diffuse peripancreatic fat stranding. There were no signs of necrosis, collections or vascular complications (Fig. 3). Therefore, the patient was concurrently diagnosed with uncomplicated acute alcoholic pancreatitis. This was managed conservatively with intravenous fluids and gradual diet upgrade. The patient's abdominal pain resolved after three days.

The patient was discharged on day five. On initial follow-up at four weeks he had moderate clinical recovery, and still became dyspnoeic on heavy lifting or several minutes of walking. He had ceased further alcohol use. Follow-up with repeat echocardiography was planned at three months, but the patient did not attend.

## Discussion and conclusions

TCM is characterised by transient left ventricular wall abnormality resulting in apical ballooning resembling an octopus trap, 'takotsubo'. Most cases of TCM are associated with a preceding stressor, and sympathetic stimulation in the setting of increased catecholamines is widely accepted to be central to its pathogenesis. Classically, this association has been with emotional trauma—that is the ‘broken heart’; however physical stressors are in fact more common. These include various physical activities, procedures, drugs, and medical conditions ranging from sepsis to lightning strike [2].

Acute pancreatitis as a trigger of TCM is a rare phenomenon. Since it was first described in 2007 [3], there have been only 11 total cases reported in the literature (Table 1). Of these, 9 were female, with ages ranging between 47 and 76 years and a median of 63 years, and 82% of cases above 50 years. This corresponds with the usual demographic of TCM, which is the post-menopausal woman [2]. Additionally, the aetiology of pancreatitis reflects that typically seen in the community, with alcohol and gallstones predominating. The timeframe for onset of pancreatitis symptoms to TCM was quite variable, ranging from hours to 7 days. Similarly, the markers of systemic inflammation were an unreliable predictor of development of TCM or its complications, with leukocytosis ranging from mild to markedly elevated.

**Table 1:** Summary of literature reporting on pancreatitis-induced TCM. Adapted from Abe et al. [4]

Authors	Sankri-Tarbichi et al. [3]	Rajani et al. [5]	Cheezum et al. [6]	Pednekar & Chandra [7]	Leubner et al. [8]	Bruenjas et al. [9]	Boulos [10]	Garbowska et al. [11]	Koop et al. [12]	Abe et al. [4]	Ashraf et al. [13]	Current case
Age (years)	56	72	76	70	76	55	47	47	63	57	64	27
Sex	Female	Female	Female	Female	Female	Male	Female	Female	Male	Female	Female	Male
Aetiology of pancreatitis	Gallstones	–	Gallstones	–	Gallstones	Alcohol	–	Alcohol	Gallstones	Alcohol	Unknown	Alcohol
Symptoms of pancreatitis	RUQ pain, nausea	Abdominal pain	N + V	RUQ pain	Epigastric pain, N + V	Epigastric pain, N + V	Epigastric pain	Epigastric pain, N + V	Epigastric pain, N + V	Abdominal pain, N + V	Epigastric pain, vomiting	Epigastric pain, N + V diaphoresis
Time to TCM	3 days	7 days	2 days	Same day	1 day	Same day	–	7 days	3 days	4 days	5 days	Same day
Systemic inflammation	–	–	WCC 23.3 × 10^9^/L	–	Mild leukocytosis	–	–	WCC 19.52 × 10^9^/L, CRP 293.8 mg/L	WCC 11.5 × 10^9^/L	WCC 14.6 × 10^9^/L	–	WCC 17.2 × 10^9^/L
Symptoms of TCM	SOB, chest pain, nausea	Chest pain	Tachypnoea, hypoxemic	Cardiac arrest	SOB, diaphoresis	Chest pain, diaphoresis, nausea	Nausea	Chest pain, SOB	Oliguria, hypotension, SOB, PEA arrest	SOB, hypoxemic	SOB	Chest pain
Troponin (ng/mL; reference < 0.02)	2.39	0.32	0.67	3.13	9.94	0.66	0.3	9.65	0.02	0.97	Elevated	1019.63
ECG	TWI V2-5	Inferolateral TWI	Lateral ST elevation	Inferior ST elevation, anterior TWI	Anteroseptal ST elevation	Generalised ST depression + TWI	Inferolateral TWI	ST elevation V2	Non-specific inferolateral T-wave changes	Diffuse ischemic TWI	Anterior ST elevation	Anterior ST elevation
Chest radiography	Pulmonary oedema	–	Pulmonary oedema, bilateral pleural effusions	–	Mild pulmonary oedema, bilateral pleural effusions	–	–	Pulmonary congestion	Acute pulmonary oedema	Pulmonary oedema	Pulmonary oedema	–
Echocardiogram or ventriculography	LVEF 25%, severe apical hypokinesia/ akinesia of left ventricle, hypercontractile base	Apical akinesis	LVEF 30%, severe apical hypokinesis + hyperdynamic basal contraction	LVEF 30%	LVEF 30–35%, hypokinetic apical left ventricle	LVEF 25%, apical ballooning, hypercontractile basal segments	Akinesis of distal anterior, lateral, inferior walls of left ventricle	LVEF 25%, apical ballooning, hypercontractile basal segments of left ventricle	LVEF 20–25%, new-onset cardiomyopathy, global hypokinesis	LVEF 40%, basal segment hyperkinesis, apical akinesis	LVEF 30–35%, mid-to-apical segments hypokinetic to akinetic	LVEF 20%, basal hyperkinesis, apical akinesis
Angiography	Normal coronary arteries	Unobstructed coronary arteries	Mild non-obstructive CAD	No obstructive atherosclerotic disease	No CAD	No obstructive CAD	Not done—myocardial nuclear stress test mildly abnormal	Normal coronary arteries	50% LAD stenosis, otherwise no obstructive CAD	Normal coronary arteries	Only luminal irregularities	Non-obstructive CAD
Treatment of TCM	Aspirin, BB, ACEi	BB, ACEi	BB, ACEi	BB, ACEi	–	Aspirin, BB, ACEi, warfarin	–	–	Left ventricular assist device	BB, ACEi	BB, ACEi	BB, ACEi
Recovery of LVEF	Yes	–	Yes	Yes	–	Yes	–	Yes	Yes	No	Yes	–
Time to recovery	10 days	–	2 weeks	6 weeks	–	3 weeks	–	10 days	3 weeks	–	6 weeks	–

*TCM* Takotsubo cardiomyopathy; *N* + *V* nausea and vomiting; *WCC* white cell count; *CRP* C-reactive protein; *SOB* shortness of breath; *PEA* pulse electrical activity; *TWI* T-wave inversion; *LVEF* left ventricle ejection fraction; *CAD* coronary artery disease; *LAD* left anterior descending; *BB* beta-blocker; *ACEi* angiotensin-converting enzyme inhibitor

Recognising TCM in the setting of pancreatitis is clinically difficult. With the exception of two cases which manifested as cardiac arrest [7, 12], TCM complicating pancreatitis results in considerable overlap in symptomology, and a patient's epigastric pain can easily mask or distract from chest pain. Formal diagnosis of TCM relies heavily on coronary angiography with left ventriculography. While multiple diagnostic criteria have been proposed, the most widely known is the Revised Mayo Clinic Criteria [2]. This requires the presence of transient left ventricular dysfunction, the absence of obstructive coronary artery disease, electrocardiographic abnormalities or troponin elevation, and the absence of pheochromocytoma and myocarditis [14]. Indeed, all previously reported cases of pancreatitis-induced TCM, and our own case, had elevated troponin, and ECG almost always demonstrated ST elevation or T-wave inversion. Chest radiography, when performed, showed features of acute pulmonary oedema. Furthermore, almost all cases demonstrated left ventricular apical hypokinesis or akinesis, basal hyperkinesis, reduced ejection fraction, and normal or non-obstructive coronary arteries (Table 1).

Further diagnostic challenge lies in the overlap of biochemical and ECG changes of TCM with those of acute coronary syndrome. Our presented case, like several others, was diagnosed presumptively as acute coronary syndrome and treated as such [5, 9]. Emergency reperfusion therapy with thrombolysis, whilst not inappropriate for this patient given the considerable delay in reaching cardiac catheterisation, is not without significant risks of bleeding and stroke [15]. However, in a centre where primary percutaneous coronary intervention was available within 90 min, a diagnosis of TCM could have been reached sooner, and the potential complications of thrombolysis could have been avoided [16]. Interestingly, there may be a role in the stable patient for early echocardiogram and subsequent computed tomography coronary angiography to reach a diagnosis of TCM, thereby avoiding the vascular complications of cardiac catheterisation [16].

The outcomes of TCM as a complication of pancreatitis appear to be good. There has been no reported deaths, even in the severe case of cardiac arrest ultimately requiring left ventricular assist device [12]. All patients, where treatments were described, were discharged on a beta-blocker and ACEi, and in those whose follow-up was reported, all but one had recovered left ventricular function, ranging from as early as 10 days up to 6 weeks. Abe et al. [4] reported the case of persistently reduced left ventricular function even at 4 months, which was hypothesised to be due to ongoing alcohol use (Table 1).

Overall, TCM is a rare, and potentially under-recognised, complication of acute pancreatitis. In a scenario where delays in diagnosis can lead to unnecessary interventions and complications, increasing awareness of this phenomenon by surgeons and physicians alike is imperative.

## Data Availability

All data generated or analysed during this study are included in this published article.

## Abbreviations

TCM: Takotsubo cardiomyopathy
ECG: Electrocardiography
ACE: Angiotensin-converting enzyme
N + V: Nausea and vomiting
WCC: White cell count
CRP: C-reactive protein
SOB: Shortness of breath
PEA: Pulseless electrical activity
TWI: T-wave inversion
LVEF: Left ventricle ejection fraction
CAD: Coronary artery disease
LAD: Left anterior descending
BB: Beta-blocker
